# One Health genomic surveillance of *Klebsiella pneumoniae* in Belgium demonstrates large genomic diversity, presence of ESBLs and carbapenemases and circulation of clones across sectors

**DOI:** 10.3389/fmicb.2026.1933141

**Published:** 2026-10-07

**Authors:** Hanne Debergh, Bert Bogaerts, Inge Van Damme, Cécile Boland, Kevin Vanneste, Koenraad van Hoorde, Pierre Bogaerts, Te-Din Huang, Marc Saulmont, Nadine Botteldoorn, Claude Saegerman, Cristina Garcia-Graells

**Affiliations:** 1 Service Foodborne Pathogens, Sciensano, Brussels, Belgium; 2 Fundamental and Applied Research for Animal and Health (FARAH) Center, University of Liège, Liège, Belgium; 3 Transversal Activities in Applied Genomics, Sciensano, Brussels, Belgium; 4 Veterinary Bacteriology Service, Sciensano, Brussels, Belgium; 5 National Reference Center for Antibiotic-Resistant Gram-Negative Bacilli, CHU UCL Namur, Yvoir, Belgium; 6 Association Régionale de Santé et d’Identification Animales, Ciney, Belgium; 7 Animal Health Care Flanders, Lier, Belgium

**Keywords:** AMR, antimicrobial resistance, *Klebsiella pneumoniae*, One Health, phylogeny, whole genome sequencing

## Abstract

*Klebsiella pneumoniae* is an important reservoir and disseminator of antimicrobial resistance (AMR) genes. Multidrug-resistant lineages, particularly extended-spectrum β-lactamase (ESBL)-producing and carbapenem-resistant strains, represent an important public health threat. This study characterized antimicrobial resistance and genomic diversity of *K. pneumoniae* across One Health compartments in Belgium and investigated genomic relatedness within and across sectors. Between 2019 and 2022, 888 presumptive *K. pneumoniae* isolates were collected from diseased animals (*n* = 311), wastewater (*n* = 299), the food chain (*n* = 178), and human clinical samples (*n* = 100). Following selection based on isolation source and AMR characteristics, 286 WGS-confirmed *K. pneumoniae* isolates were characterized for AMR and virulence determinants, plasmid replicons, population structure, and phylogenomic relatedness. Phenotypic and genotypic resistance showed high concordance for third-generation cephalosporins (93.4%) and carbapenems (89.2%). Carbapenemase genes encoding KPC, NDM, OXA-48-like, and VIM enzymes were detected in 67 isolates from humans (*n* = 56) and wastewater (*n* = 11), but not in food-chain or diseased-animal isolates. Population structure was highly diverse, with ST307, ST15, ST147, and ST11 representing the most frequently detected high-risk lineages. Thirteen novel STs were identified in 18 *K. pneumoniae* isolates. Seventeen genomic clusters were identified, including 13 within-sector and four cross-sector clusters. Notably, an ST147 cluster comprising two poultry-meat isolates and a human clinical isolate showed no SNP differences, indicating very close genomic relatedness across sectors. Overall, our findings demonstrate a genetically diverse *K. pneumoniae* population alongside the circulation of clinically relevant high-risk lineages across One Health compartments and support integrated genomic surveillance to better understand their dissemination.

## 1 Introduction

The antimicrobial resistance (AMR) crisis for the most difficult-to-treat infections in hospitals worldwide is driven by the ESKAPE pathogens: the Gram-positives *Enterococcus faecium* and *Staphylococcus aureus*, and the Gram-negatives *K. pneumoniae*, *Acinetobacter baumannii, Pseudomonas aeruginosa*, and *Enterobacter* spp. (Pendleton et al., 2013; Wyres and Holt, 2018). Typically, ESKAPE pathogens are environmental or commensal bacteria that are generally not pathogenic but can cause opportunistic infections in hospitalized or immunocompromised patients. The Gram-negative ESKAPE pathogens pose the greatest threat due to the emergence of strains resistant to multiple critical antibiotics (de Man et al., 2018). This accumulation of AMR is primarily facilitated by horizontal gene transfer (HGT) mediated by plasmids and mobile genetic elements (MGEs) (Wyres and Holt, 2018). Other mechanisms include modification of efflux pumps or permeability defects due to porin loss (Darby et al., 2023).

*Klebsiella pneumoniae* infections are associated with prolonged outbreaks, a high disease burden, and substantial mortality (Cassini et al., 2019; Murray et al., 2022). Of particular concern is the increasing prevalence of infections caused by extended-spectrum β-lactamase (ESBL)-producing and carbapenem-resistant *K. pneumoniae* (CRKp), which considerably limit available treatment options (Navon-Venezia et al., 2017; Wyres et al., 2020). The WHO Global Antibiotic Resistance Surveillance Report 2025 highlights the magnitude of this problem at the global level (World Health Organization, 2025). In 2023, more than 55% of *K. pneumoniae* causing bloodstream infections worldwide were resistant to third-generation cephalosporins (3GCs), with resistance levels exceeding 70% in the WHO African Region (World Health Organization, 2025). Carbapenem resistance is also increasingly reported, further restricting treatment options for infections caused by multidrug-resistant (MDR) *K. pneumoniae*. Importantly, the global burden of AMR is unevenly distributed, with particularly high resistance levels reported in several regions where surveillance capacity and access to adequate diagnostics and treatment may be limited (World Health Organization, 2025).

The increasing burden of resistant *K. pneumoniae* is also evident in Europe. In 2024, *K. pneumoniae* was the third most commonly reported bacterial species among invasive isolates from blood or cerebrospinal fluid in EU/EEA countries, accounting for approximately 15% of all reported invasive isolates (EARS-Net, 2024). Moreover, the estimated EU incidence of bloodstream infections caused by CRKp increased by 61.0% between 2019 and 2024 (EARS-Net, 2024). Although resistance levels in Belgium remain below the EU/EEA population-weighted mean, resistance to 3GCs and carbapenems among invasive *K. pneumoniae* isolates reached 21.0 and 1.9%, respectively, in 2024, compared with EU/EEA population-weighted mean percentages of 32.9 and 11.3%, respectively (EARS-Net, 2024). These trends emphasize that, despite comparatively lower resistance levels in Belgium, continued surveillance and prevention remain essential.

Beyond its clinical importance as a multidrug-resistant pathogen, *K. pneumoniae* plays an important role in the acquisition and dissemination of AMR determinants. Its ability to accumulate AMR genes is largely facilitated by horizontal gene transfer mediated by plasmids and other MGEs (Wyres and Holt, 2018). Several clinically important AMR genes, such as KPC-1 (Nordmann et al., 2009) and NDM-1 (Nordmann et al., 2011), were first detected in *K. pneumoniae* before subsequently being identified in other Gram-negative pathogens (Holt et al., 2015; Navon-Venezia et al., 2017), leading to its recognition as an important reservoir, amplifier and disseminator of AMR determinants (Wyres and Holt, 2018). Reflecting this clinical importance, third-generation cephalosporin-resistant and carbapenem-resistant Enterobacterales, including *K. pneumoniae*, have been included among the WHO bacterial priority pathogens (World Health Organization, 2024a).

Population genomic studies have revealed a highly diverse *K. pneumoniae* population comprising hundreds of lineages, while also identifying a limited number of globally disseminated high-risk clones that disproportionately contribute to antimicrobial-resistant infections (Wyres et al., 2020; Wyres et al., 2020). These global problem clones include CG258, CG15, CG147, CG101 and CG307 and are strongly associated with multidrug-resistant healthcare-associated infections and outbreaks (Wyres and Holt, 2016; Navon-Venezia et al., 2017; Wyres et al., 2020). In contrast, hypervirulent *K. pneumoniae* (hvKp) lineages have traditionally been associated with community-acquired infections in otherwise healthy individuals and are frequently linked to mobile virulence determinants. Major hvKp clonal groups include CG23, CG65 and CG86 (Wyres et al., 2020; Martin et al., 2023).

The One Health concept, which recognizes the interconnectedness of human, animal, and environmental health, provides a comprehensive framework for understanding and addressing the complex issue of antibiotic resistance and its transmission routes (The Food and Agriculture Organization of the United Nations, World Organization for Animal Health, World Health Organization, 2021). Previous studies have suggested that *K. pneumoniae* can persist in a broad range of reservoirs including the hospital environment (Clarivet et al., 2016; Paskova et al., 2018; Proia et al., 2018; Mathers et al., 2019), retail meat (Belmar Campos et al., 2014; Davis et al., 2015; Huizinga et al., 2019; Cardozo et al., 2021; Rodrigues et al., 2022), food-producing animals (Daehre et al., 2018; Köck et al., 2018; Bonardi and Pitino, 2019), companion animals (Debergh et al., 2022; Collineau et al., 2023) and wastewater (Galler et al., 2014; Tiwari et al., 2022; Heljanko et al., 2024). Wastewater treatment plants (WWTPs), especially those receiving hospital effluents, can represent a hotspot for high-risk bacterial pathogens, which could be discharged into surface water due to inadequate wastewater treatment (Savin et al., 2022a). *K. pneumoniae* was added in the scope of the European Antimicrobial Resistance Surveillance network in Veterinary medicine (EARS-Vet) to monitor AMR in cattle associated with mastitis (Mader et al., 2022). In general, there are only limited data on lineage distributions in non-human reservoirs, but several reports exist on the overlap between lineages isolated from human clinical isolates and other sources. For example, the global problem clones ST11 have been isolated from a dog (Debergh et al., 2022) and from poultry (Zhang et al., 2019), ST101 from pigs (De Koster et al., 2022), ST307 from a tortoise (Foster et al., 2020) and the hvKp lineage ST23 from non-human primates (Anzai et al., 2017).

In this study, we aimed to investigate *K. pneumoniae* in a One Health approach within Belgium, including clinical human isolates, isolates from diseased animals, the food chain and environmental strains. To gain insight into the antibiotic resistance profile, the virulence characteristics, and the genetic relatedness between the *K. pneumoniae* isolates, we performed antibiotic susceptibility testing (AST) and whole genome sequencing (WGS) on a subset of isolates.

## 2 Materials and methods

### 2.1 Sample and isolate collection

A total of 888 presumptive *K. pneumoniae* isolates, originating from samples from the food chain (*n* = 1,508), human clinical isolates (*n* = 100), wastewater samples (*n* = 160) and diseased animals (*n* = 311), and were collected in this study (Figure 1). More details regarding the isolation procedure of the strains can be found in Supplementary material 1. Identification of presumptive *K. pneumoniae* isolates was performed with MALDI-TOF Microflex (Bruker, Massachusetts, USA). According to the manufacturer’s recommendations, a log score value ≥ 2.0 was considered as reliable species identification.

**Figure 1:**
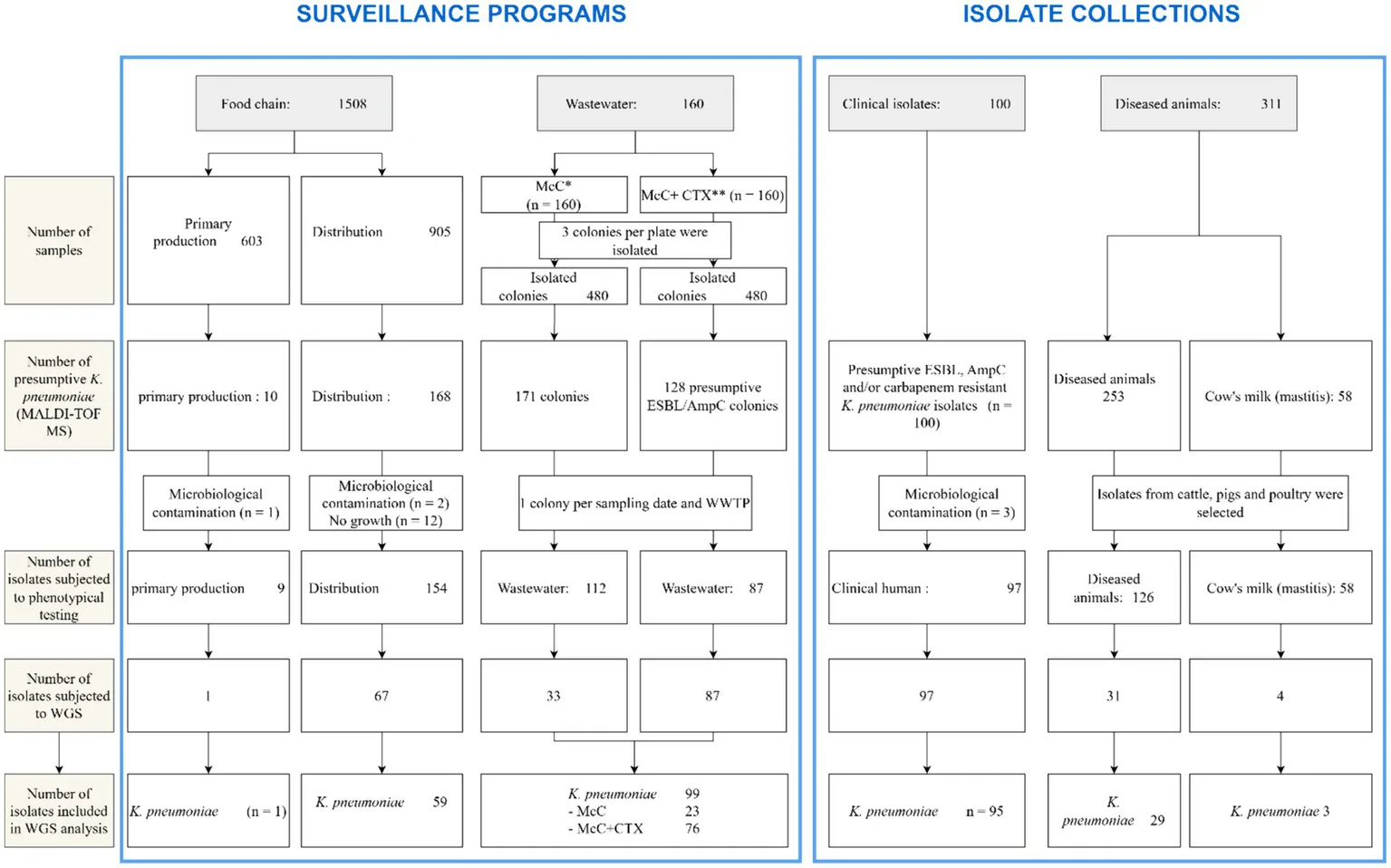
*Klebsiella pneumoniae* isolate collection flow chart. *McC, McConkey medium; **McC + CTX, McConkey + cefotaxime (1 mg/L); WWTP, Wastewater treatment plant.

### 2.2 Antimicrobial susceptibility testing

Minimal inhibitory-testing (MIC) of the isolates was conducted using broth microdilution procedure according to the reference method ISO 20776-1:2019 (International Organization for Standardization, 2019). The MIC values were interpreted according to epidemiological cutoff values that were provided by EUCAST (Official Journal of the European Union, 2020).

Broth microdilution using the Sensititre panels (Thermo Fisher, Massachusetts, USA) was performed on a selection of *K. pneumoniae* isolates (*n* = 643) (See section 4.3.1 Supplementary material 2). Susceptibility to a first panel of 14 antimicrobials (EUVSEC 3 Sensititre™, Thermo Fisher, USA) was tested for all 643 isolates. Those isolates that displayed phenotypical resistance to cefotaxime, ceftazidime and/or meropenem (*n* = 213) were assessed for susceptibility to a second panel of 10 antimicrobials (EUVSEC 2 Sensititre™, Thermo Fisher, USA). *E. coli* ATCC 25922 was used for quality control in both panels. The MIC values were interpreted according to epidemiological cutoff (ECOFF) values provided by the European Committee on Antimicrobial Susceptibility Testing (EUCAST) as described in the Commission Implementing Decision 2020/1729 for *E. coli* (Official Journal of the European Union, 2020). In the absence of ECOFF values for *K. pneumoniae*, those for *E. coli* as the *Enterobacterales* indicator, were applied (Supplementary Tables 1, 2). Isolates with a minimum inhibitory concentration (MIC) strictly higher than the EUCAST ECOFF values were referred to as ‘resistant’ rather than ‘microbiologically antimicrobial-resistant’ organisms for brevity.

### 2.3 Whole-genome sequencing and comparative genomic analyses

#### 2.3.1 Selection and flowchart for whole genome sequencing

Isolates were selected for WGS based on their source of origin and phenotypic AMR profile. Clinical isolates from diseased animals’ gastrointestinal (GI) tracts were chosen, and only one *K. pneumoniae* isolate per WWTP per sampling date was included. Only isolates recovered from food produced in Belgium were considered. Selection criteria included resistance to at least one of the following antimicrobials [classified as critically or highly important for human and veterinary medicine according to WHO classifications (World Health Organization, 2024b)]: to 3rd and 4th generation cephalosporins, carbapenems, temocillin, colistin, ciprofloxacin, azithromycin, and/or amikacin.

A total of 201 *K. pneumoniae* isolates with at least one of these resistance profiles were selected from wastewater (*n* = 110), the food chain (*n* = 60), and diseased animals (*n* = 31). Additionally, 10% of fully susceptible isolates (except to ampicillin) were included: 10 from wastewater, 5 from meat samples, 1 from primary production, 3 from veterinary sources, and 1 from milk from mastitis cases (Supplementary Figure 1). A subset of 100 ESBL/AmpC-producing and/or carbapenem-resistant clinical human strains from the NRC for antibiotic-resistant gram-negative bacteria (CHU UCL Namur Site Godinne) was included, with isolation dates and hospital locations matching the study period and locations of the WWTPs. Three out of 100 clinical isolates were excluded from further analysis due to microbiological contamination. In total, 318 isolates were sequenced (Supplementary Figure 1).

#### 2.3.2 DNA extraction, DNA library preparation, and whole-genome sequencing

Genomic DNA was extracted from all 318 *K. pneumoniae* isolates using the Maxwell cultured cells DNA extraction kit (Promega, Wisconsin, USA). DNA purity was assessed with a Nanodrop 2000 spectrophotometer (ThermoFisher Scientific, Waltham, MA, USA), and concentration was measured with a Qubit 3.0 fluorometer (Thermo Fisher Scientific, Waltham, MA, USA). Library preparation was performed with the TruSeq DNA Nano sample preparation kit (Illumina, San Diego, CA, USA), and sequencing was conducted on an Illumina NovaSeq 6,000 platform (S4 PE150 XP, Illumina, San Diego, CA, USA).

#### 2.3.3 WGS data analysis

##### 2.3.3.1 Quality control & pre-processing

Pre-processing of the sequencing data began with downsampling to approximately 100x coverage, based on the estimated genome size of 5,330,000 bp for *K. pneumoniae*, using the ‘sample’ command of seqtk v1.4.^1^ The paired-end Illumina reads are trimmed using fastp v0.23.4 (Chen, 2023) with the following options: ‘--detect_adapter_for_pe’, ‘--cut_front’, ‘--cut_front_window_size’ set to 1, ‘--cut_front_mean_quality’ set to 10, ‘--cut_tail’, ‘--cut_tail_window_size’ set to 1, ‘--cut_tail_mean_quality’ set to 10, ‘--cut_right’, ‘--cut_right_window_size’ set to 4, ‘--cut_right_mean_quality’ set to 20, and ‘--length_required’ set to 40. The processed reads were *de novo* assembled using SPAdes v3.15.5 (Prjibelski et al., 2020) with the ‘--cov_cutoff’ parameter set to 10 and the ‘--isolate’ option enabled. Contigs smaller than 1 kb were removed using the seq function of seqtk v1.4 (see text footnote 1). Assembly metrics were calculated using QUAST 5.2.0, with the processed FASTQ files and assembled contigs as input, and the *Klebsiella pneumoniae* subsp. *pneumoniae* HS11286 genome as reference (RefSeq accession NC_016845.1). Processed reads were mapped to the assembly using Bowtie2 v2.5.1 (Langmead and Salzberg, 2012) with the ‘--end-to-end’ and ‘--sensitive’ options. The median depth was determined using the ‘depth’ command from samtools v1.17 (Li et al., 2009) with the ‘-a’ option enabled.

##### 2.3.3.2 Isolate characterization

All assemblies were analyzed using Kleborate v2.3.2 (Lam et al., 2021) with the ‘--all’ option. Species identification was performed using the Kleborate tool, which also provides standardized resistance and virulence scores for *K. pneumoniae* isolates based on the presence of key antimicrobial resistance genes (ARGs) and virulence loci identified through WGS (Lam et al., 2021). Detection of AMR mutations and genes was performed using AMRFinder+ v3.11.26 (Feldgarden et al., 2021) with the 2021-12-21.1 database. Virulence genes were identified using the Kleborate tool (Lam et al., 2021). The ‘mob_recon’ command of MOB-suite v3.1.4 (Robertson and Nash, 2018) was used to screen for MGEs, and contigs with detected resistance and virulence genes were cross-referenced with MOB-suite output to predict their potential association with MGEs. Sequence types (STs) were determined using MiST v1.0.0 (Bogaerts et al., 2025a) and the MLST scheme from the Institut Pasteur Paris BIGSdb instance (Diancourt et al., 2005; Bogaerts et al., 2021) (accessed on the 24th of May 2026). Novel STs were obtained by submitting the corresponding alleles and profiles into the database.

##### 2.3.3.3 Phylogenomic analysis

Core genome MLST (cgMLST) was performed using the scheme from the Institut Pasteur Paris BIGSdb instance 51 (accessed on August 18, 2022) using the methodology detailed in the previous section. A minimum spanning tree was constructed from the filtered allele matrix after removing datasets with less than 90% of loci detected and loci present in less than 75% of samples. GrapeTree v2.2 was used to construct the phylogeny with the ‘MSTreeV2’ method. For each ST where at least two isolates had fewer than **10** allele differences, an additional SNP-based phylogenomic analysis was conducted (Supplementary material 3). This involved mapping processed reads to a close reference genome of the same ST or clonal complex, with the selected reference genomes listed in Supplementary material 4.

Sequences other than the chromosome were removed from the reference genome FASTA files prior to the SNP analysis. The PACU v0.0.3 ‘map_to_ref’ script was used with default options to map reads, and SNP-based phylogenies were constructed using PACU v0.0.3 with the ‘--use-mega’ option enabled (Bogaerts et al., 2024).

##### 2.3.3.4 Comparison between phenotype and genotype for carbapenem and 3GC resistance

The agreement between phenotypic antimicrobial resistance and genomic detection of antimicrobial resistance determinants was evaluated for carbapenem and third-generation cephalosporin resistance. Phenotypic carbapenem resistance was defined based on susceptibility testing results for imipenem and/or meropenem, while the corresponding genotypic resistance determinant was defined as the presence of an acquired carbapenemase gene identified by Kleborate. Phenotypic third-generation cephalosporin resistance was defined by resistance to cefotaxime and/or ceftazidime. The corresponding genotypic resistance determinants included acquired ESBL genes, acquired carbapenemase genes, and the AmpC β-lactamase gene DHA-1. For each resistance category, two-by-two contingency tables were constructed to compare phenotypic and genotypic classifications. Sensitivity, specificity, and overall concordance were calculated as measures of genotype–phenotype agreement.

The agreement between phenotypic antimicrobial resistance and genomic detection of corresponding antimicrobial resistance determinants was evaluated for carbapenems and 3GCs. Phenotypic carbapenem resistance was defined as resistance to meropenem and/or imipenem, while the corresponding genotypic determinant was defined as the presence of an acquired carbapenemase gene identified by Kleborate. Phenotypic 3GC resistance was defined as resistance to cefotaxime and/or ceftazidime. Corresponding genotypic determinants included acquired ESBL genes, acquired carbapenemase genes, and the acquired AmpC β-lactamase gene *bla*DHA-1. For each antimicrobial category, a two-by-two contingency table was constructed comparing the phenotypic classification with the presence or absence of the corresponding genotypic determinants. Phenotypic susceptibility testing was used as the reference classification. Sensitivity was calculated as the proportion of phenotypically resistant isolates carrying a corresponding genotypic resistance determinant [true positives/(true positives + false negatives)], whereas specificity was calculated as the proportion of phenotypically susceptible isolates lacking the corresponding determinant [true negatives/(true negatives + false positives)]. Overall concordance was calculated as the proportion of isolates for which the phenotypic and genotypic classifications agreed [(true positives + true negatives)/total number of isolates].

### 2.4 Statistical analysis

The proportion of resistant isolates were calculated per sector and were visualized using ggplot2 (Wickham, 2016). To visualize the combination of resistance against different antibiotics, the package UpSetR was used (Conway et al., 2017). All analyses were performed using R version 4.2.3. (R Core Team, 2023).

## 3 Results

### 3.1 Occurrence of *Klebsiella pneumoniae* in the food chain and in wastewater

Given that our isolates were obtained conveniently from other projects with different objectives and corresponding sampling strategies, comparison of prevalence or detection levels across the sectors is not possible.

In primary production, *K. pneumoniae* was detected in 1.7% (10/603) of samples. Detection was observed in 1.4% (3/214) of fecal samples at farm level, 1.7% (2/115) of fecal samples from fattening pigs, and 2.0% (3/147) of fecal samples from broiler chickens, while no *K. pneumoniae* was detected in fecal samples from veal calves (0/111). In addition, *K. pneumoniae* was detected in 12.5% (2/16) of raw bulk-tank milk samples.

In fresh meat, *K. pneumoniae* was detected in 18.6% (168/905) of samples. Detection rates were 16.6% (70/422) in fresh pork, beef, or turkey meat, 22.9% (22/96) in complete poultry carcasses, and 19.6% (76/387) in cut poultry with or without skin.

In wastewater samples, we observed a 69.4% (111/160) prevalence of *K. pneumoniae* on McConkey agar (McC) and a 54.4% (87/160) prevalence of presumptive ESBL- and/or AmpC- producing *K. pneumoniae* isolates using McConkey agar supplemented with cefotaxime [1 mg/L; McC + CTX].

### 3.2 Antimicrobial susceptibility testing

AST using the EUVSEC 3 panel was performed on 643 *K. pneumoniae* isolates, including 199 wastewater, 97 human clinical, 163 food-chain and 184 diseased-animal isolates (Table 1). As expected for *K. pneumoniae*, resistance to ampicillin was observed in nearly all tested isolates across sectors (96.0–100.0%). Resistance to most other antimicrobials varied considerably between sectors.

**Table 1:** Number of *K. pneumoniae* isolates and percentage of phenotypical resistance against each antimicrobial included in EUVSEC 3 for the four different sectors according to the ECOFF values (EUCAST, 2026).

Antimicrobial	Wastewater (*n* = 199)		Human clinical* (*n* = 97)		Food chain (*n* = 163)		Diseased animals (*n* = 184)		Total* (*n* = 643)	
	*n*	%	*n*	%	*n*	%	*n*	%	*n*	%
Amikacin	1	0.5	16	16.5	1	0.6	0	0.0	18	2.8
Ampicillin	191	96.0	97	100.0	159	97.5	182	98.9	629	97.8
Azithromycin	37	18.6	39	40.2	15	9.2	18	9.8	109	17.0
Chloramphenicol	25	12.6	41	42.3	18	11.0	18	9.8	102	15.9
Ciprofloxacin	74	37.2	86	88.7	40	24.5	14	7.6	214	33.3
Cefotaxime	90	45.2	90	92.8	3	1.8	7	3.8	190	29.5
Colistin	10	5.0	11	11.3	14	8.6	9	4.9	44	6.8
Gentamicin	45	22.6	39	40.2	6	3.7	8	4.3	98	15.2
Meropenem	13	6.5	82	84.5	1	0.6	0	0.0	96	14.9
Nalidixic Acid	60	30.2	79	81.4	29	17.8	7	3.8	175	27.2
Sulfamethoxazole	79	39.7	75	77.3	57	35.0	30	16.3	241	37.5
Ceftazidime	83	41.7	79	81.4	7	4.3	8	4.3	177	27.5
Tetracycline	53	26.6	56	57.7	59	36.2	33	17.9	201	31.3
Tigecycline	0	0.0	6	6.2	0	0.0	0	0.0	6	0.9
Trimethoprim	72	36.2	72	74.2	45	27.6	30	16.3	219	34.1

*n*, number of tested *K. pneumoniae* isolates; * Human clinical isolates were part of a selection based on presumptive EBSL-producing or carbapenem resistant *K. pneumoniae* isolates possibly resulting in higher resistance rates toward other antimicrobials.

Among wastewater isolates, resistance was most frequently observed for cefotaxime (45.2%), ceftazidime (41.7%), sulfamethoxazole (39.7%), ciprofloxacin (37.2%) and trimethoprim (36.2%). Resistance to meropenem was detected in 6.5% of wastewater isolates, while resistance to colistin and amikacin was observed in 5.0 and 0.5%, respectively.

High resistance percentages were observed among the selected human clinical isolates, particularly for cefotaxime (92.8%), ciprofloxacin (88.7%), meropenem (84.5%), ceftazidime and nalidixic acid (both 81.4%), sulfamethoxazole (77.3%) and trimethoprim (74.2%). Resistance to colistin and tigecycline was observed in 11.3 and 6.2% of the clinical isolates, respectively. These percentages should be interpreted in the context of the selection strategy, as the clinical isolates were selected based on presumptive ESBL production and/or carbapenem resistance.

Resistance was generally less frequent among isolates from the food chain and diseased animals. In food-chain isolates, excluding intrinsic ampicillin resistance, the highest resistance percentages were observed for tetracycline (36.2%), sulfamethoxazole (35.0%), trimethoprim (27.6%) and ciprofloxacin (24.5%). Cefotaxime and ceftazidime resistance were detected in 1.8 and 4.3% of food-chain isolates, respectively, while meropenem resistance was detected in one isolate (0.6%). Among isolates from diseased animals, resistance was most frequently observed for tetracycline (17.9%), sulfamethoxazole and trimethoprim (both 16.3%). Resistance to cefotaxime and ceftazidime was detected in 3.8 and 4.3% of isolates, respectively, whereas no meropenem-resistant isolates were identified.

Based on their resistance profiles in the EUVSEC 3 panel, 213 isolates were selected for further characterization using the EUVSEC 2 panel, comprising 93 wastewater, 97 human clinical, 13 food-chain and 10 diseased-animal isolates (Table 2). As expected from this targeted selection, high percentages of resistance to extended-spectrum cephalosporins were observed. Cefotaxime resistance ranged from 53.8% among selected food-chain isolates to 96.8% among wastewater isolates, while ceftazidime resistance ranged from 53.8 to 89.2%. Cefepime resistance was observed in 95.7% of wastewater and 93.8% of clinical isolates and in 53.8 and 70.0% of the selected food-chain and diseased-animal isolates, respectively.

**Table 2:** Number of selected *K. pneumoniae* isolates (based on observed resistance profile in the EUVSEC 3 panel) and percentage of phenotypical resistance against each antimicrobial included in EUVSEC 2 for the four different sectors according to the ECOFF values (EUCAST, 2026).

Antimicrobial	Wastewater (*n* = 93)		Human clinical* (*n* = 97)		Food chain (*n* = 13)		Diseased animals (*n* = 10)		Total* (*n* = 213)	
	*n*	%	*n*	%	*n*	%	*n*	%	*n*	%
Cefepime	89	95.7	91	93.8	7	53.8	7	70.0	194	91.1
Cefotaxime	90	96.8	90	92.8	7	53.8	7	70.0	194	91.1
cefotaxime + clavulanic acid	19	20.4	82	84.5	2	15.4	2	20.0	105	49.3
cefoxitine	28	30.1	65	67.0	3	23.1	3	30.0	99	46.5
Ertapenem	52	55.9	91	93.8	4	30.8	4	40.0	151	70.9
Imipenem	12	12.9	59	60.8	0	0.0	0	0.0	71	33.3
Meropenem	13	14.0	82	84.5	0	0.0	0	0.0	95	44.6
Ceftazidime	83	89.2	79	81.4	7	53.8	8	80.0	177	83.1
Ceftazidime + clavulanic acid	20	21.5	67	69.1	2	15.4	2	20.0	91	42.7
Temocillin	21	22.6	83	85.6	2	15.4	2	20.0	108	50.7

*n*, number of tested *K. pneumoniae* isolates; *Human clinical isolates were part of a selection based on presumptive EBSL-producing and/or carbapenem resistant *K. pneumoniae* isolates possibly resulting in higher resistance rates toward other antimicrobials.

Among the selected wastewater isolates, resistance to ertapenem was observed in 55.9%, whereas resistance to imipenem and meropenem was considerably less frequent (12.9 and 14.0%, respectively). Among the selected clinical isolates, resistance to ertapenem, meropenem and imipenem was observed in 93.8, 84.5 and 60.8%, respectively. No imipenem or meropenem resistance was detected among the selected food-chain or diseased-animal isolates. Resistance to temocillin was observed in 22.6% of wastewater isolates and 85.6% of clinical isolates, compared with 15.4 and 20.0% of selected food-chain and diseased-animal isolates, respectively.

### 3.3 Comparison between phenotypic and genotypic resistance

Phenotypic carbapenem resistance, defined as resistance to imipenem and/or meropenem, was compared with the genomic detection of acquired carbapenemase genes. Overall concordance was 89.2% (255/286), with a sensitivity of 68.8% and specificity of 99.5% (Table 3). Acquired carbapenemase genes were detected in 66/96 (68.8%) phenotypically carbapenem-resistant isolates. Thirty phenotypically resistant isolates lacked an acquired carbapenemase gene, whereas only one phenotypically susceptible isolate carried a carbapenemase gene (*bla*_OXA-48_). Concordance was highest among diseased-animal (100%) and food-chain isolates (98.3%), although carbapenem resistance was absent or rare in these sectors, and was 94.9% among wastewater and 73.7% among clinical isolates.

**Table 3:** Comparison of carbapenem resistance phenotype and related genotype.

Matrix	*N*	pR	pS	pRgR	pSgR	pSgS	pRgS	Concordance	Se	Sp
Diseased animal	32	0	32	0	0	32	0	100%	NA	100
Food chain	60	1	59	0	0	59	1	98.3%	0	100
Human	95	81	14	56	0	14	25	73.7%	69	100
Wastewater	99	14	85	10	1	84	4	94.9%	71	99
Overall	286	96	190	66	1	189	30	89%	69	99

*N*, number of strains; pR, number of strains with phenotypic resistance (IMI and/or MERO); pS, number of strains phenotypically susceptible; pRgR, phenotypically resistance and presence of acquired carbapenemase genes; pSgR, phenotypically susceptible and presence of acquired carbapenemase genes; pSgS, phenotypically susceptible and no acquired carbapenemase genes; pRgS, phenotypically resistant without acquired carbapenemase genes; Se, sensitivity; Sp, specificity.

Among the 30 phenotypically carbapenem-resistant isolates without an acquired carbapenemase gene, 16 (53.3%) carried predicted alterations in OmpK35 and/or OmpK36. Several of these isolates additionally carried other acquired β-lactamases, including OXA-1, TEM-1D and DHA-1, or chromosomal SHV variants.

For third-generation cephalosporins, phenotypic resistance was defined as resistance to cefotaxime and/or ceftazidime. Genotypic resistance determinants comprised acquired ESBL genes, acquired carbapenemase genes and/or *bla*DHA-1. Overall concordance between phenotype and genotype was 93.4% (267/286), with a sensitivity of 89.7% and specificity of 100% (Table 4). Of 185 phenotypically resistant isolates, 166 (89.7%) carried at least one of the predefined resistance determinants, whereas 19 (10.3%) did not. None of the 101 phenotypically susceptible isolates carried one of these determinants. Concordance was highest among wastewater isolates (99.0%), followed by diseased-animal (93.8%), food-chain (91.7%) and clinical isolates (88.4%).

**Table 4:** Comparison of third generation cephalosporin resistance phenotype and related genotype.

Matrix	*N*	pR	pS	pRgR	pSgR	pSgS	pRgS	Concordance	Se	Sp
Diseased animal	32	8	24	6	0	24	2	93.8%	75	100
Food chain	60	7	53	2	0	53	5	91.7%	29	100
Human	95	89	6	78	0	6	11	88.4%	88	100
Wastewater	99	81	18	80	0	18	1	99%	99	100
Overall	286	185	101	166	0	101	19	93.4%	90	100

*N*, number of strains; pR, number of strains with phenotypic resistance (Cefotaxime and/or Ceftazidime); pS, number of strains phenotypically susceptible; pRgR, phenotypically resistance and presence of acquired acquired ESBL and/or AmpC genes; pSgR, phenotypically susceptible and presence of acquired ESBL and/or AmpC genes; pSgS, phenotypically susceptible and no acquired ESBL and/or AmpC genes; pRgS, phenotypically resistant without acquired ESBL and/or AmpC genes; Se, sensitivity; Sp, specificity.

### 3.4 Genomic characterization using WGS

Whole-genome sequencing was performed on 318 isolates initially identified as *K. pneumoniae*. A total of 17 isolates did not meet the quality criteria and were removed from the analysis. Genomic species identification confirmed 286 isolates as *K. pneumoniae*, whereas 15 isolates were reclassified as other members of the *K. pneumoniae* species complex, including *K. quasipneumoniae* subsp. *similipneumoniae* (*n* = 10), *K. quasipneumoniae* subsp. *quasipneumoniae* (*n* = 2), and *K. quasivariicola* (*n* = 3). Subsequent genomic analyses were restricted to the 286 confirmed *K. pneumoniae* isolates.

#### 3.4.1 Diversity in AMR genes and virulence

Heatmaps displaying resistance and virulence scores according to Kleborate, stratified by isolation source, are shown in Figure 2. Most isolates (192/286; 67.1%) had a virulence score of 0, while 94/286 (32.9%) had a virulence score ≥1. No isolate had the maximum virulence score of 5. Six isolates (6/286; 2.1%) had a virulence score of 4, comprising three human clinical and three diseased-animal isolates, while another six isolates (6/286; 2.1%) had a virulence score of 3, comprising one human clinical and five diseased-animal isolates (Table 5). Notably, all eight diseased-animal isolates with virulence scores ≥3 originated from pigs and represented eight different sequence types (ST17, ST25, ST35, ST37, ST37-1LV, ST45, ST113, and ST530). No wastewater isolate had a virulence score ≥3, and no food-chain isolate had a virulence score ≥2.

**Figure 2:**
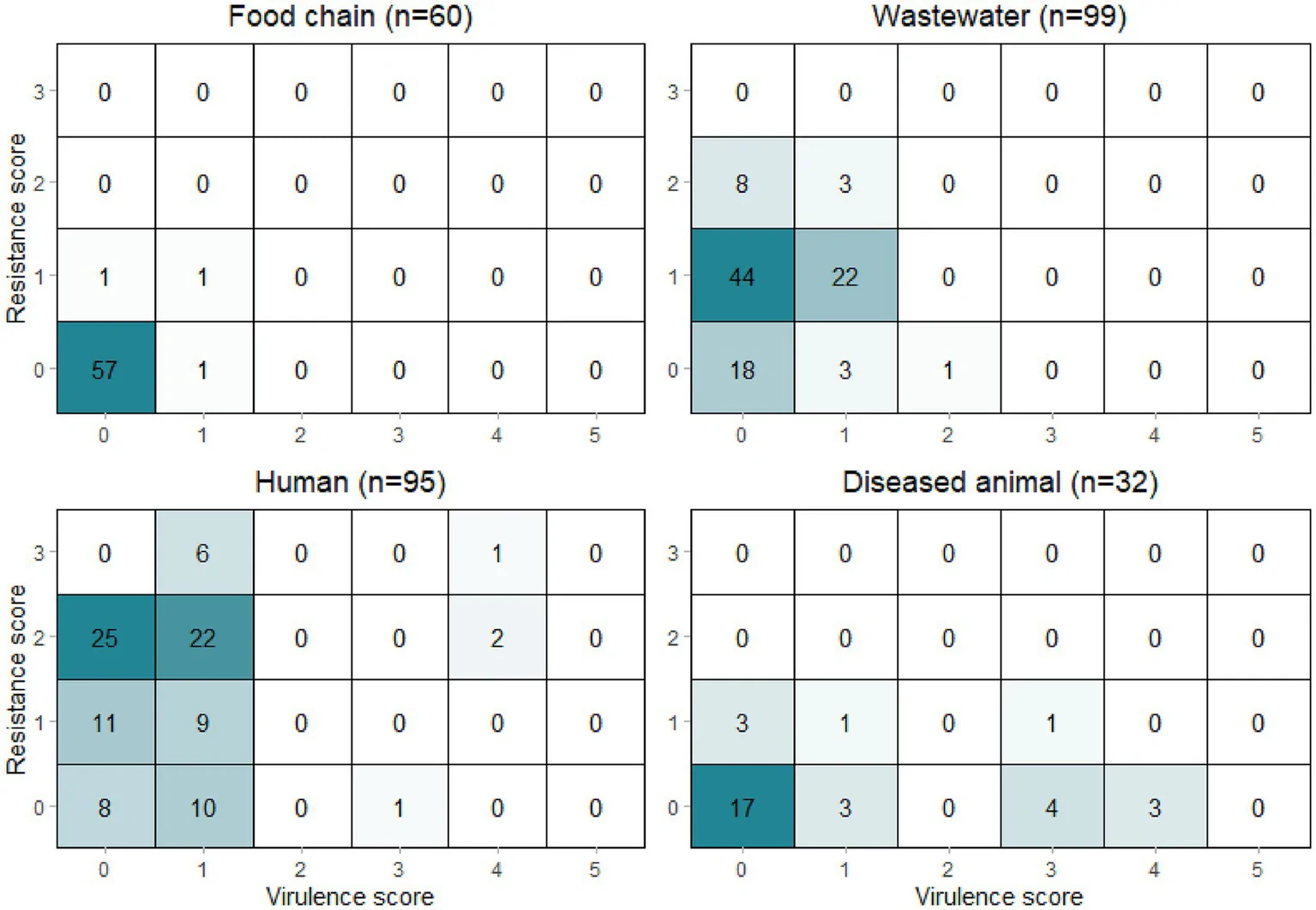
Heatmap displaying resistance and virulence scores, and the number of isolates with those characteristics stratified by isolation matrix. A total of 286 confirmed *K. pneumoniae* isolates were included from the food chain (*n* = 60), diseased animals (*n* = 32), wastewater (*n* = 99), and human isolates (*n* = 95). Virulence score: 0 = no yersiniabactin, colibactin and aerobactin; 1 = yersiniabactin only; 2 = yersiniabactin and colibactin; 3 = aerobactin only; 4 = aerobactin with yersiniabactin (no colibactin); 5 = yersiniabactin, colibactin and aerobactin. Resistance score: 0 = no ESBL/carbapenemase/colistin resistance determinants detected; 1 = ESBL; 2 = carbapenemase; 3 = carbapenemase plus colistin resistance. Resistance scores and virulence scores were obtained by Kleborate (Lam et al., 2021).

**Table 5:** Isolates with a virulence score of 4 (according to Kleborate) with their accompanying virulence genes.

Isolate	Source	ST	Yersiniabactin	Aerobactin	*rmpADC*	*rmpA2*
S22FP03118	Pig	ST 113	*ybt 8; ICEKp3*	*iuc3*	-	-
S22FP03119	Pig	ST 45	*ybt 10; ICEKp4*	*iuc3*	-	-
S22FP03161	Pig	ST 530	*ybt 9; ICEKp3*	*iuc3*	-	-
S23FP03518	Human	ST 2096	*ybt 14; ICEKp5*	*iuc1*	-	*rmpA2*_8–60%
S23FP03545	Human	ST 101	*ybt 9; ICEKp3*	*iuc1*	-	-
S23FP03806	Human	ST 709	*ybt 9; ICEKp3*	*iuc1*	*rmp 1; KpVP-1*	*rmpA2_*6*-47%

No colibactin and salmochelin genes were detected.

The ‘*rmpADC*’ and ‘*rmpA2*’ columns indicate operon and gene encoding hypermucoviscosity. A dash (‘-’) indicates that the corresponding genomic element was not detected. The ‘*rmpADC*’ column reports the *rmpADC* locus sequence type, together with the associated lineage and mobile genetic element. The rmpA2 column lists the detected rmpA2 allele, with an asterisk (*) denoting an imperfect match. Truncated genes are represented by a percentage, which indicates the length of the amino acid sequence relative to that of the full-length protein (calculated from the start codon).

None of the isolates carried the salmochelin locus (*iro*), while colibactin was detected in only one wastewater isolate. Aerobactin showed a distinct source-associated distribution: *iuc3* was exclusively detected in eight isolates from diseased pigs, whereas *iuc1* was exclusively detected in four human clinical isolates. Three isolates (3/286; 1.0%) carried a truncated *rmpA2* gene. Phenotypic assays to confirm hypermucoidy or hypervirulence were not performed.

Convergence of antimicrobial resistance and virulence determinants was observed in three human clinical isolates with a virulence score of 4 and resistance scores ≥2. These isolates belonged to ST2096, ST101, and ST709 and carried *iuc1* together with yersiniabactin. The ST2096 and ST709 isolates additionally carried truncated *rmpA2*. The three isolates carried *bla*OXA-232, *bla*KPC-3, and *bla*NDM-5, respectively.

Resistance scores differed markedly according to isolation source. A resistance score of 3, indicating the presence of a carbapenemase together with genetic determinants associated with colistin resistance, was observed exclusively among human clinical isolates (*n* = 7). These isolates belonged to internationally recognized high-risk lineages ST11, ST15, and ST101. A detailed overview of their STs, ESBL and carbapenemase genes, outer-membrane protein mutations, and *mgrB* alterations is provided in Table 6. None carried a known plasmid-mediated *mcr* gene, and phenotypic colistin resistance was confirmed in two isolates (S23FP03817 and S23FP03837).

**Table 6:** Human isolates displaying a resistance score of 3 with their acquired ESBL and carbapenemase genes, the mutations in the outer membrane proteins and the mutations in the *mgrB* gene conferring colistin resistance.

Isolate	ST	ESBL genes	Carbapenemase genes	*OmpK* mutations	Col mutations*
S23FP03545	ST 101	-	*bla*_KPC-3_	*OmpK35*-17%; *OmpK36TD*	*mgrB* -0%
S23FP03563	ST 15	*bla*_CTX-M-15_	*bla*_OXA-48_	*OmpK35*-70%	*mgrB* -6%
S23FP03804	ST 11	*bla*_CTX-M-15_	*bla*_NDM-1_;*bla*_OXA-181_	*OmpK36GD*	*mgrB* -60%
S23FP03813	ST 11	*bla*_CTX-M-15_	*bla*_NDM-1_;*bla*_OXA-181_	*OmpK36GD*	*mgrB* -60%
S23FP03817^#^	ST 15	-	*bla*_OXA-48_	*OmpK35*-70%	*mgrB* -6%
S23FP03837^#^	ST 15	*bla*_CTX-M-15_	*bla*_OXA-48_	*OmpK35*-70%	*mgrB* -6%
S23FP03848	ST 11	*bla*_CTX-M-15_	*bla*_NDM-1_;*bla*_OXA-181_	*OmpK36GD*	*mgrB* -60%

*The column ‘col mutations’ shows if the *mgrB* gene is missing or truncated. Truncations are expressed as % amino acid length from the start codon. Note if MgrB is present and not truncated then nothing about them will be reported in the ‘Col’ column [wild-type MgrB protein is associated with full susceptibility to colistin (Poirel et al., 2015)]. GD, Glycine-Aspartate; TD, Threonine-Aspartate. ^#^ Phenotypical colistin resistance as tested by broth microdilution (Official Journal of the European Union, 2020).

A resistance score of 2, corresponding to carbapenemase production in the absence of identified genetic colistin resistance determinants, was observed in 49 human clinical isolates (51.6%) and 11 wastewater isolates (11.1%), but was not detected among food-chain or diseased-animal isolates. Resistance score 1, reflecting ESBL production in the absence of carbapenemase or genetic colistin resistance determinants, predominated among wastewater isolates (*n* = 66; 66.7%). In contrast, a resistance score of 0, indicating the absence of clinically important acquired AMR determinants included in the Kleborate scoring system, predominated among isolates from the food chain (*n* = 57/59; 96.6%) and diseased animals (*n* = 28/33; 84.8%).

#### 3.4.2 Number of acquired AMR gene per isolate

The number of acquired ARGs per isolate was assessed according to isolation source. Clinical isolates carried a median of 10 acquired ARGs per isolate (range: 0–17), followed by wastewater isolates with a median of 8 (range: 0–24), food-chain isolates with a median of 5 (range: 0–16), and isolates from diseased animals with a median of 2 (range: 0–7). Considerable variation in ARG burden was observed within sectors, particularly among wastewater and food-chain isolates.

#### 3.4.3 Distribution of carbapenemase genes

Carbapenemase genes encoding KPC, NDM, OXA-48-like, and VIM enzymes were detected in 67/286 (23.4%) *K. pneumoniae* isolates, comprising 56 human clinical isolates and 11 wastewater isolates (Supplementary Figure 3). No carbapenemase genes were detected among isolates from the food chain or diseased animals.

OXA-48-like carbapenemases (*bla*_OXA-48_, *bla*OXA-181, and *bla*OXA-232) were the most frequently detected carbapenemases and were present in 53/67 (79.1%) carbapenemase-positive isolates. *bla*_OXA-48_ was the predominant variant (n = 45), followed by *bla*_OXA-181_ (n = 5) and *bla*OXA-232 (n = 2); one additional isolate co-carried *bla*_OXA-48_ and *bla*NDM-1. Overall, 40/53 (75.5%) isolates carrying an OXA-48-like carbapenemase also carried the ESBL gene *bla*CTX-M-15. Five human clinical isolates co-carried an OXA-48-like carbapenemase with either *bla*NDM-1 or *bla*NDM-5.

KPC carbapenemases were detected in six isolates (6/67; 9.0%), including five carrying *bla*KPC-3 and one carrying *bla*KPC-2. Four of the six KPC-producing isolates originated from wastewater and two from human clinical samples. The wastewater isolates belonged to ST258 (*bla*KPC-3), ST317-1LV (*bla*KPC-3), ST34 (*bla*KPC-3), and ST11 (*bla*KPC-2), whereas the clinical isolates belonged to ST512 and ST101, both carrying *bla*KPC-3. In addition, *bla*VIM-1 was detected in two clinical isolates, belonging to ST307 and ST556.

Given that carbapenem non-susceptibility can also arise from mutations in the outer-membrane proteins (*OmpK35* and/or *OmpK36*) in combination with the expression of ESBL and/or acquired AmpC β-lactamases, all strains were screened for known mutations in these genes. Mutations in *OmpK35* and/or *OmpK36* were found in 16 isolates without a carbapenemase gene, of which 12 carried the ESBL gene *bla*_CTX-M-15_. Among these 12, eight isolates were non-susceptible to all tested carbapenem antibiotics, while the remaining four were resistant to meropenem and ertapenem but susceptible to imipenem.

#### 3.4.4 Plasmid replicons

The distribution of plasmid replicons according to isolation source is shown in Table 7. In total, 945 plasmid replicon sequences were detected across the 286 *K. pneumoniae* isolates, of which 379 (40.1%) were detected in wastewater isolates, 337 (35.7%) in human clinical isolates, 132 (14.0%) in food-chain isolates, and 97 (10.3%) in isolates from diseased animals. IncFIB(K) was the most frequently detected replicon (*n* = 182), followed by IncFII(K) (*n* = 144) and IncR (*n* = 80). These three replicon types were detected across all four One Health compartments and together accounted for 43.0% (406/945) of all replicon detections (Table 7).

**Table 7:** Plasmid replicon characterization.

Locus	Diseased animal	Food chain	Human	Wastewater	Total
IncFIB(K)_1	23	21	61	77	182
IncFII(K)_1	13	13	53	65	144
IncR_1	11	16	27	26	80
IncL_1	0	0	38	7	45
IncHI1B(pNDM-MAR)_1	2	7	17	16	42
IncFIA(HI1)_1	2	7	14	19	42
Col440I_1	1	2	11	22	36
IncFIB(K)(pCAV1099-114)_1	7	5	11	12	35
repB(R1701)_1	0	4	18	12	34
ColRNAI_1	7	0	17	6	30
Others	31	57	70	117	275
Total	97	132	337	379	945

The top 10 detected plasmid replicons and their respective frequency of detection per matrix.

In contrast, IncL showed a more restricted distribution. Of the 45 IncL replicons detected, 38 (84.4%) occurred in human clinical isolates and seven (15.6%) in wastewater isolates, while IncL was not detected among food-chain or diseased-animal isolates. IncHI1B(pNDM-MAR) was detected across all four compartments, although more frequently in human (*n* = 17) and wastewater (*n* = 16) isolates than in food-chain (*n* = 7) or diseased-animal isolates (*n* = 2). A similar distribution was observed for IncFIA(HI1). Col440I was predominantly detected in wastewater isolates (22/36; 61.1%).

IncX3, which was not among the 10 most frequently detected replicons shown in Table 7, was detected in five clinical isolates and one isolate from a diseased animal. Three of the IncX3-positive clinical isolates carried carbapenemase genes, including *bla*OXA-181, *bla*NDM-7, and *bla*NDM-5.

#### 3.4.5 Population structure and phylogeny

Considerable genetic diversity was observed among the 286 WGS-confirmed *K. pneumoniae* isolates, comprising 125 distinct STs, of which 76 (60.8%) were represented by a single isolate. In addition, 13 novel STs were identified among 18 *K. pneumoniae* isolates and submitted to the Institut Pasteur BIGSdb database. Novel STs were recovered from multiple isolation sources (wastewater [*n* = 8], food chain [*n* = 7], diseased animals [*n* = 2], clinical [*n* = 1]) further illustrating the genetic diversity of the collection. More details are provided in Table 8. Despite this high diversity, several STs occurred repeatedly. The most frequently identified lineages were ST307 (*n* = 21; 7.3%), ST15 (*n* = 18; 6.3%), ST147 (*n* = 13; 4.5%), and ST11 (*n* = 12; 4.2%), followed by ST37 and ST39 (*n* = 8 each), ST14 and ST870 (*n* = 6 each), and ST45, ST405, and ST152 (*n* = 5 each) (Figure 3).

**Table 8:** Metadata of the 17 novel sequence types.

Isolates	ST	Source type	Host	Isolation year	City	Country
S20FP07792	7655	Animal	Chicken	2020	Unknown	Belgium
S21FP02019	7656	Animal	Pig	2021	Unknown	Belgium
S21FP02030	7657	Animal	Chicken	2021	Unknown	Belgium
S21FP02032	7656	Animal	Cow	2021	Unknown	Belgium
S21FP02035	7656	Animal	Chicken	2021	Unknown	Belgium
S21FP02223	7658	Animal	Cow	2021	Unknown	Belgium
S21FP02371	7658	Animal	Cow	2021	Unknown	Belgium
S22FP03024	7659	Animal	Cow	2021	Unknown	Belgium
S22FP03092	7660	Animal	Pig	2020	Unknown	Belgium
S22FP05828	7661	Wastewater	Wastewater	2021	Brussels North	Belgium
S22FP05833	7661	Wastewater	Wastewater	2021	Brussels North	Belgium
S22FP05842	7662	Wastewater	Wastewater	2021	Brussel South	Belgium
S22FP05851	7661	Wastewater	Wastewater	2021	Brussels North	Belgium
S22FP05894	7663	Wastewater	Wastewater	2021	Brussel South	Belgium
S22FP05918	7664	Wastewater	Wastewater	2021	Aalst	Belgium
S22FP05919	7665	Wastewater	Wastewater	2021	Brussel South	Belgium
S22FP05942	7666	Wastewater	Wastewater	2022	Grimbergen	Belgium
S22FP05971	7667	Wastewater	Wastewater	2022	Leuven	Belgium
S22FP05981	7668	Wastewater	Wastewater	2022	Brussels North	Belgium
S22FP05991	7669	Wastewater	Wastewater	2022	Leuven	Belgium
S23FP03528	7670	Human	Human	2021	Zwijnaarde	Belgium
S23FP03535	7671	Human	Human	2021	Assebroek	Belgium
S21FP02771	7658	Food chain	Chicken	2021	Unknown	France

ST, Sequence type; ST, Sequence type.

**Figure 3:**
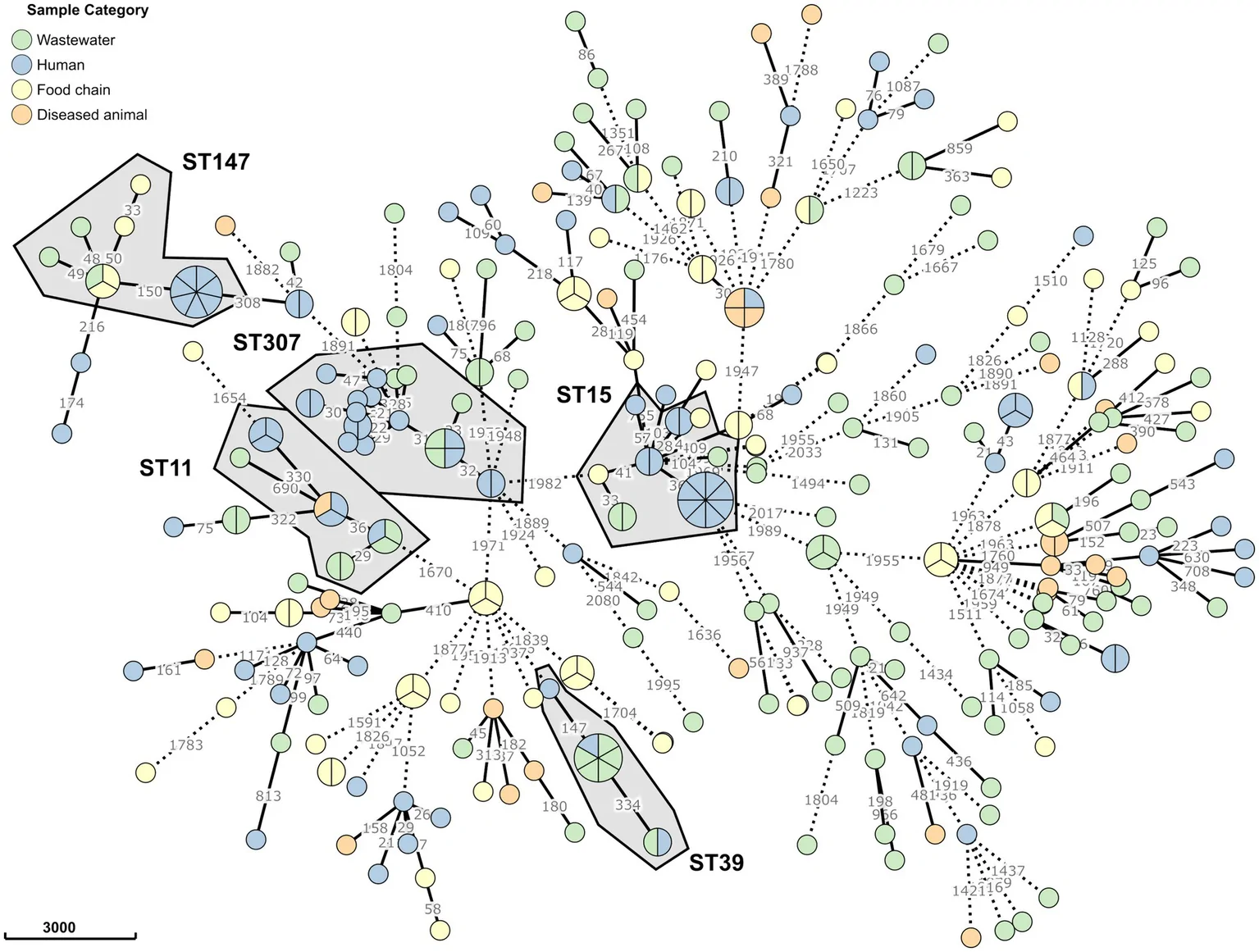
Minimum spanning tree based on core genome multilocus sequence typing (cgMLST) of 286 *K. pneumoniae* isolates, colored by sample category as indicated in the legend. Branches were collapsed at ≤ 10 allele differences, and branches exceeding 1,000 allele differences were truncated at 1,000 and are indicated by a striped line. Branch lengths and the scale bar represent the number of cgMLST allele differences on a logarithmic scale. The cgMLST scheme comprises 2,358 loci in total. The five most frequently observed STs are grouped together and labeled.

The four most frequently detected STs showed distinct distributions across isolation sources. ST307 was predominantly identified among clinical isolates (16/21), with the remaining five isolates recovered from wastewater. ST15 was detected among clinical (13/18), wastewater (3/18), and food-chain (2/18) isolates, while ST147 was similarly recovered from clinical (7/13), wastewater (3/13), and food-chain (3/13) sources. ST11 was identified among clinical (6/12) and wastewater (5/12) isolates and in one isolate from a diseased animal.

Several other recurrent STs were also detected across multiple One Health compartments. ST37 was identified in clinical (*n* = 4), wastewater (*n* = 3), and diseased-animal (*n* = 1) isolates; ST870 occurred among diseased animals (*n* = 3), food-chain isolates (*n* = 2), and a clinical isolate (*n* = 1); ST14 was detected in clinical (*n* = 3), food-chain (*n* = 2), and diseased-animal (*n* = 1) isolates; and ST405 was recovered from wastewater (*n* = 2), clinical (*n* = 2), and diseased-animal (*n* = 1) sources. Additional STs shared between two sectors included ST39, ST152, and ST323 among clinical and wastewater isolates; ST42, ST469, and ST280 among food-chain and wastewater isolates; and ST661 and ST29 among diseased-animal and wastewater isolates (Supplementary Figure 4).

Overall, the population structure was characterized by a large number of infrequently detected STs alongside a smaller number of recurrent lineages occurring across different isolation sources. High-resolution assessment of the genetic relatedness of isolates belonging to recurrent STs was subsequently performed using cgMLST and SNP-based phylogenomic analysis.

#### 3.4.6 Cluster analysis of *Klebsiella pneumoniae* within and across sectors

Cluster analysis within and across sectors was performed for STs in which at least two isolates differed by fewer than five cgMLST alleles, using SNP-based phylogenomic analysis. In total, 17 clusters were identified, of which 13 occurred within the same sector (Table 9) and four involved isolates from different sectors (Table 10; Figure 3).

**Table 9:** Cluster analysis of *K. pneumoniae* isolates within sectors.

ST	Isolates	SNP differences	Source	Location	Date of isolation
ST11	S23FP3804	8–9	Clinical ^#^	Genk	04/11/2021
	S23FP3813				23/11/2021
	S23FP3848				18/02/2022
	S22FP5848	5	Wastewater	Harelbeke	22/04/2021
	S22FP5976				02/08/2022
ST307	S23FP3533	3	Clinical ^#^	Brussels	05/07/2021
	S23FP3542				03/08/2021
	S23FP3816	6	Clinical ^#^	Brussels	25/11/2021
	S23FP3818				16/12/2021
	S22FP5835	1	Wastewater	Brugge	13/04/2021
	S22FP5844			Ghent	20/04/2021
ST15	S23FP3549	14	Clinical ^#^	Tienen	08/09/2021
	S23FP3565				14/10/2021
	S23FP3825	13	Clinical ^#^	Leuven	06/01/2022
	S23FP3851				25/02/2022
	S23FP3837	3–6	Clinical ^#^	Leuven	31/01/2022
	S23FP3563				13/10/2022
	S23FP3817				07/12/2022
ST147	S23FP3539	7	Clinical ^#^	Oostende	22/07/2021
	S23FP3842			Veurne	09/02/2022
ST39	S22FP5980	1–16	Wastewater	Brussels South	22/02/2022
	S22FP5982			Ghent	22/02/2022
	S23FP5972			Brussels South	02/08/2022
ST870	S20FP7796	13	Food (poultry meat)	Belgium *	05/10/2020
	S21FP1932				02/03/2021
	S22FP3076	1–7	Diseased animal (Pig)	Belgium *	10/12/2019
	S22FP3129				04/02/2020
	S22FP3128				04/02/2020
ST152	S23FP3530	7–10	Clinical^#^	Waregem	24/06/2021
	S23FP3532			Roeselare	30/06/2021
	S23FP3537			Aardooie	08/07/2021

*No details regarding the isolation location were available. ^#^No information about the specific wards was available. ST, Sequence type; SNP, single nucleotide polymorphism.

**Table 10:** Cluster analysis of *K. pneumoniae* isolates across sectors.

ST	Isolates	SNP differences	Source	Location	Date of isolation
11	S22FP3181	2–13^¥^	Diseased animal (dog)	n/a, BE*	12/06/2020
	S23FP3823		Clinical^#^	Huy, BE	04/01/2022
	S23FP3822		Clinical^#^	Huy, BE	04/01/2022
	S22FP5847	10–15	Wastewater	Leuven, BE	22/04/2021
	S22FP5864		Wastewater	Leuven, BE	19/08/2021
	S23FP3810		Clinical^#^	Geel, BE	18/11/2022
147	S21FP3491	0	Food (poultry meat)	n/a, BE*	13/04/2021
	S21FP3503		Food (poultry meat)	n/a, BE*	14/04/2021
	S22FP5824		Clinical^#^	Leuven, BE	29/04/2021
42	S20FP8496	8	Food (beef meat)	n/a, BE*	12/10/2020
	S22FP5920		Wastewater	Leuven, BE	14/12/2021

*No specific details regarding the isolation location were available. ^#^No information about the specific wards were available. ^¥^Between the clinical isolates a distance of 2 SNPs was observed.

Within-sector clusters included clinical isolates belonging to ST11, ST15, ST147, ST152 and ST307, wastewater isolates belonging to ST11, ST39 and ST307, isolates from diseased animals belonging to ST870, and food-chain isolates belonging to ST870. Pairwise SNP distances within these clusters ranged from 1 to 16 SNPs. Several clusters contained isolates recovered from different geographical locations or at different time points. For example, two ST307 wastewater isolates recovered from WWTPs in Bruges and Ghent differed by only one SNP.

Four clusters comprising isolates from different One Health sectors were identified. ST11 was represented in two cross-sector clusters: one comprising human clinical and wastewater isolates and another comprising human clinical isolates and an isolate from a diseased animal. Pairwise SNP distances within these ST11 clusters ranged from 2 to 15 SNPs. Cross-sector clustering was also observed for ST147 and ST42. The ST147 cluster comprised two isolates recovered from poultry meat on 13 and 14 April 2021 and a clinical isolate recovered in Leuven on 29 April 2021. Remarkably, no SNP differences were detected between these isolates. The ST42 cluster comprised an isolate from beef meat and an isolate recovered from wastewater, which differed by eight SNPs. Overall, these results demonstrate the presence of closely related *K. pneumoniae* isolates not only within individual sectors, but also across human, animal, food-chain and environmental compartments.

## 4 Discussion

Surveillance studies that apply a One Health framework—integrating data from the food chain, diseased animals, environmental sources, and clinical settings—remain limited for *Klebsiella pneumoniae* (Wyres et al., 2020). This species is characterized by considerable genetic heterogeneity and a remarkable capacity to acquire and disseminate ARGs (Wyres et al., 2020). In this study, we investigated the prevalence of *K. pneumoniae* isolates and resistance profiles, focusing particularly on ESBL-producing and carbapenem-resistant strains, across sectors including food production, diseased animals, wastewater, and human clinical isolates.

Interpretation of the results should be made cautiously, given methodological differences across sectors. For instance, isolation strategies varied: MacConkey (McC) agar was used for food chain and animal samples, while a selective McC + cefotaxime (CTX) medium was employed for wastewater samples to select for ESBL producers. Human clinical isolates were preselected based on suspected ESBL or carbapenem resistance, introducing a strong selection bias that likely overestimates the resistance and virulence gene burden in clinical and wastewater-derived isolates. Resistance screening methods were not used for samples originating from diseased animals. These methodological differences may also have influenced phylogenomic relationships and cluster detection outcomes.

Additionally, by using the McC medium, we may have missed some isolates by overgrowth of *E. coli*, possibly leading to an underrepresentation of *K. pneumoniae* in the primary production sector. Species identification further complicates surveillance accuracy; reliance on MALDI-TOF MS introduces limitations in resolving members of the *K. pneumoniae* species complex (KpSC). In our study, 15 isolates initially identified as *K. pneumoniae* by MALDI-TOF were subsequently reclassified as other KpSC species (*K. quasivariicola, Klebsiella quasipneumoniae subsp. Quasipneumoniae* and *Klebsiella quasipneumoniae subsp. Similipneumoniae*) by WGS, underscoring the importance of genomic confirmation for species-level identification (Rodrigues et al., 2018; Thorpe et al., 2022).

We detected *K. pneumoniae* in 18.6% of fresh meat samples, which is lower than reported in other studies: 60% KpSC in chicken meat in a European study (Rodrigues et al., 2022), 47% in retail turkey, chicken, and pork products from the United states (Davis et al., 2015), and 46.7% in calf and chicken meat samples from Turkey (Gundogan et al., 2011). Differences could be explained by differences in the methodology applied. Furthermore, large variations in prevalence of *K. pneumoniae* per country are expected, as shown in the study by Rodrigues et al. (2022).

ESBL-producing *K. pneumoniae* carrying the *bla*_CTX-M-15_ gene was detected in only 0.6% (1/168) of the samples, a rate comparable to the 1.3% observed in a European study (Rodrigues et al., 2022). This isolate showed resistance to ertapenem (MIC = 0.06 μg/mL), but not to meropenem or imipenem, which aligns with other studies (ANSES, 2022; Martak et al., 2024). No ARGs or outer membrane porin mutations were present that could explain the phenotypical ertapenem resistance.

In our study, the highest phenotypical AMR rates detected (excluding ampicillin due to intrinsic resistance) in *K. pneumoniae* from the food chain were tetracycline (36.2%), sulfamethoxazole (35.0%), and trimethoprim (27.6%) resistance. These findings reflect recent reports of antimicrobial sales for use in food-producing animals in various European countries from 2010 to 2022 (European Medicines Agency, 2023).

*Klebsiella pneumoniae* was also detected in 69.4% of wastewater samples, with 54.4% of isolates positive for presumptive ESBL- or AmpC-producing isolates. Cefotaxime resistance was observed in 45.2% of isolates, comparable to rates reported by Ludden et al. (2020), with high resistance also found to ceftazidime (41.7%), sulfamethoxazole (39.7%), ciprofloxacin (37.2%), and trimethoprim (36.2%). Over half of the wastewater isolates from McC + CTX displayed ertapenem resistance, and a subset of isolates also showed meropenem and imipenem resistance, although differences in isolation methods may partly explain variations with previous studies displaying higher ertapenem resistance prevalence in wastewater isolates (Savin et al., 2022a, 2022b).

Beyond the sector-specific resistance frequencies, we also assessed the concordance between phenotypic resistance and genomic detection of corresponding AMR determinants. Overall, phenotypic resistance showed good agreement with the genomic detection of corresponding acquired AMR determinants, although concordance differed between antimicrobial classes. Agreement was particularly high for third-generation cephalosporins (93.4%), with a sensitivity of 89.7% and specificity of 100%. Importantly, none of the phenotypically susceptible isolates carried an acquired ESBL, carbapenemase or *bla*DHA-1 determinant included in the genotypic definition. Nineteen phenotypically resistant isolates lacked these predefined determinants. Several nevertheless carried other β-lactamases, including OXA-1, TEM-1D and chromosomal SHV variants, indicating that phenotypic resistance cannot always be predicted solely from the presence of acquired ESBL, AmpC or carbapenemase genes. Differences in β-lactamase expression, permeability and other regulatory or resistance mechanisms not captured by the genomic prediction may contribute to these discrepancies (Debergh et al., 2022; Li et al., 2023). Concordance was somewhat lower for carbapenem resistance (89.2%), primarily because 30 of 96 phenotypically resistant isolates lacked an acquired carbapenemase gene. This does not necessarily represent unexplained resistance, as carbapenem resistance in *K. pneumoniae* can arise through non-carbapenemase mechanisms (Debergh et al., 2022; Thacharodi et al., 2026). Notably, predicted alterations in OmpK35 and/or OmpK36 were identified in 16/30 (53.3%) of these carbapenemase-negative resistant isolates, and several additionally carried other β-lactamases, including OXA-1, TEM-1D and DHA-1. Reduced outer-membrane permeability resulting from OmpK35/OmpK36 alterations, particularly in combination with ESBL or AmpC production, may increase carbapenem MICs in the absence of an acquired carbapenemase. The remaining discordant isolates may involve changes in expression or permeability that cannot be inferred reliably from the resistance-gene screening applied here (Thacharodi et al., 2026). Conversely, only one phenotypically carbapenem-susceptible isolate carried an acquired carbapenemase gene, *bla*_OXA-48_. This finding is biologically plausible because OXA-48 may confer relatively low-level carbapenem resistance, particularly in the absence of additional permeability defects or other resistance mechanisms (Bakthavatchalam et al., 2016). Together, these findings demonstrate that WGS-based detection of acquired resistance determinants correlated well with phenotypic susceptibility overall, while also highlighting the importance of alternative resistance mechanisms and the continued value of phenotypic antimicrobial susceptibility testing.

The high prevalence of multidrug-resistant *K. pneumoniae* in wastewater highlights the role of WWTPs as potential reservoirs and dissemination points for clinically relevant resistance. Phylogenomic analysis revealed clustering between clinical and wastewater isolates, suggesting possible human-environmental transmission links. Carbapenemase-producing *K. pneumoniae* was detected in surface water and the hospital environment, where hospital effluent plays a vital role in the dissemination of carbapenemase-producing *K. pneumoniae* (Crettels et al., 2026). Similar trends were described by Crettels et al. for ESBL-producing *E. coli* in hospital and WWTP settings (Crettels et al., 2024). Despite a general reduction of antibiotic-resistant bacteria by WWTPs (Wang et al., 2020), the persistence of resistant strains and genes in treated effluents demonstrates that conventional treatments could remain insufficient to fully eliminate these threats (Kizny Gordon et al., 2017; Müller et al., 2018; Surleac et al., 2020; Wang et al., 2020; Kehl et al., 2022; Savin et al., 2022b; Cimen et al., 2023). Caution is necessary as influent and effluent bacterial content might differ.

A study from Austria reported two MDR *K. pneumoniae* ST985 isolates with identical cgMLST profiles sampled 200 km apart, illustrating the potential for long-distance survival in river water (Müller et al., 2018). Similarly, our analysis identified a cluster of ST307 isolates, separated by only one SNP, from wastewater samples collected a week apart at WWTPs in Ghent and Bruges (50 km apart). This may reflect either environmental persistence or a shared upstream source. Notably, *Klebsiella* isolates in wastewater retained clinically relevant resistance traits, with at least 77 isolates in our collection exhibiting a resistance score ≥1. These findings suggest that dissemination of 3GC-resistant and carbapenem-resistant *K. pneumoniae* to human and animal populations remains a significant risk (Kehl et al., 2022).

Most *K. pneumoniae* isolates exhibited a virulence score of 0, while 32.9% (94/286) had a virulence score ≥1. Virulence scores ≥3 were restricted to human clinical isolates and isolates from diseased pigs. Notably, all eight pig isolates with virulence scores ≥3 carried *iuc3* and belonged to different STs, suggesting that the occurrence of *iuc3* in the porcine isolates was not attributable to expansion of a single lineage. Previous studies have similarly reported *iuc3* in *K. pneumoniae* from pigs (Kaspersen et al., 2023) and pork meat (Crippa et al., 2023), whereas *iuc1*, which in our collection was restricted to human clinical isolates, is commonly associated with human hypervirulent *K. pneumoniae* lineages (Lam et al., 2018; Zhu et al., 2023).

Of particular concern, three clinical isolates combined a virulence score of 4 with resistance scores ≥2 and belonged to three distinct lineages (ST2096, ST101, and ST709), carrying *bla*OXA-232, *bla*KPC-3, and *bla*NDM-5, respectively. This illustrates the convergence of clinically important AMR and virulence determinants in genetically distinct *K. pneumoniae* lineages. Such convergence may occur through the acquisition and recombination of resistance and virulence plasmids and represents an important clinical concern (Lepuschitz et al., 2019; Rocha et al., 2022; Cimen et al., 2023; Russo et al., 2024). Nevertheless, Kleborate virulence scores represent genotypic predictions and do not necessarily equate to a hypervirulent phenotype. Phenotypic characterization would therefore be required to confirm hypervirulence in these isolates.

The burden of acquired AMR genes varied considerably within and between One Health compartments. Clinical isolates carried the highest median number of acquired ARGs (median 10; range 0–17), followed by wastewater isolates (median 8; range 0–24), whereas lower median numbers were observed among food-chain (median 5; range 0–16) and diseased-animal isolates (median 2; range 0–7). This was accompanied by a high diversity of plasmid replicons, particularly among wastewater isolates, further supporting the relevance of this compartment for the circulation of mobile resistance determinants. A total of 945 plasmid replicon sequences were detected, with 75.8% occurring in clinical or wastewater isolates. IncFIB(K), IncFII(K), and IncR were the most frequently detected replicons and occurred across all four One Health compartments, whereas IncL showed a markedly different distribution, being detected exclusively in clinical and wastewater isolates and predominantly in the former. This distribution is noteworthy given the well-established association between IncL/M plasmids and *bla*_OXA-48_ dissemination. However, plasmid reconstruction from short-read sequencing is inherently limited, and the co-occurrence of ARGs and plasmid replicons within an isolate does not demonstrate their physical association. Long-read sequencing would therefore be required to resolve the genetic context and transfer potential of clinically relevant resistance determinants.

Assessing public health risks from environmental AMR reservoirs remains challenging without integrated large-scale WGS-based surveillance (Holmes et al., 2016; Stanton et al., 2020; Thorpe et al., 2022). Although wastewater-based epidemiology has primarily focused on resistant *E. coli* (Hutinel et al., 2019; Pärnänen et al., 2019; Huijbers et al., 2020), its application to *K. pneumoniae* remains limited, despite its potential for early outbreak detection and population-level monitoring (Sims and Kasprzyk-Hordern, 2020).

Carbapenemase genes were restricted to human clinical and wastewater isolates, with OXA-48-like enzymes accounting for the majority (79.1%) of carbapenemase-positive isolates. Of particular interest, 75.5% of isolates carrying an OXA-48-like carbapenemase also carried *bla*CTX-M-15, illustrating the frequent co-occurrence of carbapenem and extended-spectrum cephalosporin resistance determinants. OXA-48-like carbapenemases, particularly OXA-48, are widely disseminated among *K. pneumoniae* in European healthcare settings and are frequently associated with successful high-risk lineages. Their detection in wastewater in the present study indicates that clinically relevant carbapenemase-producing *K. pneumoniae* are also present outside the clinical setting and may enter wastewater systems through human-associated sources.

The detection of KPC-producing *K. pneumoniae* in wastewater was particularly noteworthy. Four of the six KPC-positive isolates originated from wastewater and included ST258 carrying *bla*KPC-3 and ST11 carrying *bla*KPC-2, both representing internationally recognized high-risk lineages. In contrast, the ST512/*bla*KPC-3 and ST101/*bla*KPC-3 combinations were detected among clinical isolates. The occurrence of high-risk clones carrying clinically important carbapenemases in wastewater supports its potential value as a complementary matrix for AMR surveillance. However, their presence in wastewater should not be interpreted as evidence that wastewater constitutes an independent reservoir or source of transmission, as these organisms may reflect shedding from colonized or infected individuals. Integration of wastewater surveillance with contemporaneous clinical genomic data would be required to determine whether wastewater can reliably capture the circulation and emergence of carbapenemase-producing lineages in the population.

Population structure analysis using MLST and cgMLST revealed considerable genetic diversity among the *K. pneumoniae* isolates, with 125 distinct STs identified among 286 isolates. Despite this diversity, 22.4% of the isolates belonged to recognized high-risk clones ST11, ST15, ST147, and ST307. Several of these clones were identified across multiple One Health sectors. ST307 occurred in clinical and environmental samples, ST15 and ST147 were detected in clinical, environmental, and food-chain isolates, while ST11 was found across human, environmental, and veterinary sources. The globally disseminated problem clones ST258 and ST512 were both detected once and carried *bla*KPC-3, consistent with their well-established association with carbapenem resistance (Campos-Madueno et al., 2022). The occurrence of high-risk clones across distinct ecological compartments indicates that these lineages are not restricted to healthcare settings and supports the importance of considering their circulation from a One Health perspective.

Phylogenomic analysis identified multiple clusters involving high-risk clones, both within and across sectors. ST15 was particularly notable as the second most frequently detected lineage (*n* = 18) and was recovered from clinical, wastewater, and food-chain sources. Carbapenemase genes were detected in 12 ST15 isolates, predominantly *bla*_OXA-48_, which occurred in both clinical and wastewater isolates. Within the clinical sector, three ST15 clusters harboring *bla*_OXA-48_ were identified, with isolates differing by ≤14 SNPs. Although epidemiological data were insufficient to confirm direct transmission, their close genomic relatedness is compatible with recent or ongoing circulation within healthcare settings. This is consistent with the international importance of ST15 as a high-risk healthcare-associated lineage frequently associated with ESBLs and carbapenemases. Recent genomic surveillance in Spanish ICUs similarly identified ST15 isolates carrying *bla*_OXA-48_ together with *bla*VIM-1 and *bla*CTX-M-15 and demonstrated closely related isolates from different patients (Loiodice et al., 2025), further illustrating the capacity of this lineage to acquire clinically important resistance determinants and circulate within healthcare settings. Several OXA-48-producing ST15 isolates in our collection additionally shared the KL24/O1-O2v1 surface antigen profile. However, the occurrence of ST15 with similar resistance and surface antigen profiles in both clinical and wastewater compartments does not by itself demonstrate epidemiological linkage, emphasizing the need for high-resolution genomic comparison combined with detailed epidemiological metadata when investigating potential One Health connections. Closely related isolates were also observed for ST307, with two clinical *bla*_OXA-48_-positive isolates from Brussels differing by only three SNPs, suggesting local circulation of a closely related lineage. Long-term persistence of *K. pneumoniae* clones has previously been demonstrated in healthcare environments (Carlsen et al., 2023), further underscoring the need for integrated genomic surveillance.

Closely related isolates were also detected in wastewater. Two ST307 isolates recovered from WWTPs in Ghent and Bruges 1 week apart differed by only one SNP, indicating the presence of an almost identical lineage at geographically distinct locations. Furthermore, two ST11 isolates recovered 4 months apart from the same WWTP were closely related, which may indicate either persistence of this lineage within the wastewater system or repeated introduction from a common or recurrent source, although the relative contribution of persistence and repeated introduction cannot be distinguished from our data.

Evidence of closely related *K. pneumoniae* lineages was also observed in the food-chain and veterinary sectors. Two ST870 isolates recovered from poultry meat 5 months apart clustered together, while three closely related ST870 isolates were identified among diseased pigs. These findings suggest either persistence or repeated introduction of specific *K. pneumoniae* lineages within food-production and animal reservoirs. Similar observations have been reported in poultry production, including clonal dissemination of colistin-resistant *K. pneumoniae* among chicken flocks (Mourão et al., 2023). Although the overall *K. pneumoniae* population in our study was highly diverse, the occurrence of closely related isolates within animal and food-chain compartments demonstrates that individual lineages can persist or disseminate within these settings.

Of particular interest were four genomic clusters spanning different One Health sectors. The strongest genomic link was observed for ST147, for which two isolates recovered from poultry meat on consecutive days and a human clinical isolate recovered in Leuven approximately 2 weeks later showed no SNP differences. The absence of detectable SNP differences indicates extremely close genomic relatedness and is compatible with a recent common origin. However, the lack of detailed metadata on the geographical origin of the poultry meat, processing facilities, and potential epidemiological connections precluded identification of a direct transmission route. Contamination during slaughter or meat processing could represent one possible pathway, as cross-contamination of carcasses with ESBL-producing Enterobacterales through shared processing environments and water has previously been demonstrated (Projahn et al., 2019). Nevertheless, alternative explanations, including circulation of the same clone through a broader common source, cannot be excluded. Given the clinical importance and global dissemination of ST147, including its frequent association with carbapenemases and healthcare-associated outbreaks (Wyres et al., 2020), the identification of an indistinguishable lineage in poultry meat and a human clinical sample warrants further investigation.

Additional cross-sector clusters further supported genomic overlap between human, animal, food-chain, and environmental compartments. An ST42 isolate recovered from fresh beef meat clustered with a wastewater isolate, with a difference of eight SNPs despite being collected approximately 14 months apart. Although their genomic relatedness suggests circulation of related ST42 lineages across sectors, the temporal interval and absence of detailed source information prevent further inference regarding epidemiological linkage. Similarly, an ST11 isolate recovered from a diseased companion animal was closely related to two human clinical ST11 isolates from the same hospital, suggesting a potential epidemiological connection between human and companion-animal populations. Closely related *K. pneumoniae* strains have previously been reported in humans and companion animals, supporting the potential for interspecies exchange at this interface (Marques et al., 2019; Garcia-Fierro et al., 2022; Røken et al., 2022). Recent genomic characterization of animal-derived *K. pneumoniae* from Germany similarly identified several human-associated STs, including ST147, ST101, ST37, ST20, ST29, ST323 and ST45, further demonstrating that clinically relevant lineages are not restricted to human populations (Bassiouny et al., 2026). Although direct contact or transmission could not be established in the present study, these observations highlight the importance of including companion animals in genomic AMR surveillance.

Finally, a cross-sector ST11 cluster comprising two wastewater isolates from Leuven and a clinical isolate from Geel showed pairwise differences of 10–15 SNPs. While the greater genomic distance provides weaker evidence for a recent epidemiological connection than observed for the ST147 cluster, the finding is consistent with the broader circulation of ST11 across human and environmental compartments. Together with the detection of ST11, ST15, ST147, and ST307 across multiple sectors, these findings demonstrate that clinically relevant high-risk *K. pneumoniae* lineages are not confined to hospitals but can also be detected in animals, the food chain, and wastewater.

Overall, our findings reveal a highly diverse *K. pneumoniae* population alongside the circulation of several internationally recognized high-risk clones across One Health compartments. The identification of closely related isolates within and, importantly, across sectors provides evidence of genomic connectivity between human, animal, food-chain, and environmental reservoirs, although the direction and mechanisms of transmission cannot be established from genomic data alone. Wastewater emerged as a particularly relevant compartment, harboring several clinically important high-risk clones and genetically related isolates detected in other sectors, supporting its potential value as a complementary source for population-level AMR surveillance. The cross-sector clusters involving poultry meat, companion animals, wastewater, and human clinical isolates further emphasize the need for integrated One Health surveillance. However, limited metadata and differences in sampling and isolation strategies between sectors restricted epidemiological interpretation. Future surveillance should therefore combine standardized sampling with detailed epidemiological metadata and genomic characterization to better resolve transmission pathways and assess the contribution of non-human reservoirs to the emergence and dissemination of high-risk *K. pneumoniae*.

## Data Availability

All tools to perform the bioinformatics analysis are freely available for non-commercial use at https://galaxy.sciensano.be (Bogaerts et al., 2025b). The WGS data are available in the NCBI (https://www.ncbi.nlm.nih.gov) under accession number BioProject accession PRJNA1262391.

## Footnotes

1
https://github.com/lh3/seqtk
