# Supplementary material for: One Health genomic surveillance of Klebsiella pneumoniae in Belgium demonstrates large genomic diversity, presence of ESBLs and carbapenemases and circulation of clones across sectors
Source: Front Microbiol. 2026 Oct 7;17:1933141. doi: 10.3389/fmicb.2026.1933141 (PMC13683611; doi:10.3389/fmicb.2026.1933141)
Supplement: Supplementary file 1 [file Data_Sheet_1.zip › Supplementary material/Supplementary figure 3_revised2.docx]

Supplementary figure 3 - Distribution of carbapenemase encoding genes (KPC, NDM, OXA-48-like and VIM) detected in carbapenem resistant isolates (according to EUCAST ECOFF values of meropenem >0.125 mg/L and imipenem >1 mg/L)
