# Supplementary material for: One Health genomic surveillance of Klebsiella pneumoniae in Belgium demonstrates large genomic diversity, presence of ESBLs and carbapenemases and circulation of clones across sectors
Source: Front Microbiol. 2026 Oct 7;17:1933141. doi: 10.3389/fmicb.2026.1933141 (PMC13683611; doi:10.3389/fmicb.2026.1933141)
Supplement: Supplementary file 1 [file Data_Sheet_1.zip › Supplementary material/Supplementary figure 4.docx]

Supplementary figure 4 - Number of isolates per observed sequence type. Results are stratified by origin (orange = diseased animal; yellow = food chain, blue = human, green = wastewater); Only STs detected in two or more isolates are included in this figure.
