# Supplementary material for: One Health genomic surveillance of Klebsiella pneumoniae in Belgium demonstrates large genomic diversity, presence of ESBLs and carbapenemases and circulation of clones across sectors
Source: Front Microbiol. 2026 Oct 7;17:1933141. doi: 10.3389/fmicb.2026.1933141 (PMC13683611; doi:10.3389/fmicb.2026.1933141)
Supplement: Supplementary file 1 [file Data_Sheet_1.zip › Supplementary material/Supplementary material 1_140826.docx]

Supplementary material 1 - Sample collection

Sample collection included convenient sampling from different sampling collections and surveillance activities. Sample collection from the food chain and from human clinical samples were part of National AMR surveillance in Belgium, whereas samples from diseased animals were collected specifically for this project. Wastewater samples were conveniently collected as part of the SARS-COV-2 surveillance program in wastewater in Belgium during the COVID pandemic. Details of each sampling campaign is provided below.

1.1 **The food chain**

A total of 1508 samples from the annual Belgian AMR surveillance on bacteria isolated from the food chain were collected by the Federal Agency for the Safety of the Food Chain (FASFC) from September 2020 to April 2021, as part of a voluntary monitoring according to EFSA ^96^. In total, 87.2% (1315/1508) originated from national production, whereas the rest of the samples (12.8%; 193/1508) were imported. The samples included 905 samples (60%) from fresh meat (distribution sector) and 603 samples (40%) from feces and raw milk (primary production). Fresh meat included fresh pork, beef, or turkey meat (n=422), whole poultry carcasses (n=96), and cut poultry with or without skin (n=387). In the primary production, fecal samples were taken at slaughterhouses from veal calves (n=111), fattening pigs (n=115), and broiler chickens (n=147), and at the farm level, 214 fecal samples of veal calves (younger than 7 months) or from poultry were collected. Additionally, 16 raw cow’s milk samples were taken directly from bulk tanks at farms.

Samples were processed using the EURL-AR protocol to isolate ESBL-, AmpC-, and carbapenemase-producing *E. coli* ^97^. This involved incubating 1g ± 0.1 g of fecal samples, 25g ± 0.5 g of meat samples or 25ml ± 0.5 ml of milk samples in 9ml, 225ml and 225ml buffered peptone water (BPW), respectively, at 37°C for 18-22 hours, followed by streaking 10 µl onto MacConkey (McC) agar plates and incubating again at 37°C for 18-22 hours. The agar plates were then sent to the National Reference Laboratory Antimicrobial Resistance (NRL-AMR, Sciensano), where three to five presumptive *K. pneumoniae* colonies were subcultured on nutrient agar (NA) for species identification using MALDI-TOF MS and antimicrobial susceptibility testing (AST) (see below). Colonies identified as *Klebsiella* spp. were stored in glycerol at -80°C for further analysis. All *K. pneumoniae* isolates from the food chain were subjected to AST. A total of 15 isolates were excluded due to microbiological contamination or loss of the isolate.

1.2 **Wastewater**

From April 2021 to March 2022, *K. pneumoniae* was isolated from ten wastewater treatment plants (WWTPs) in Flanders and Brussels serving a total of 2,536,616 inhabitants across ten locations in Belgium at the time of sampling. The isolation of composite 24h influent wastewater samples was performed weekly during four non-consecutive months, covering each season. In total, 160 composite wastewater samples were analysed. The WWTPs processed both community and hospital wastewater. Samples were diluted 1:1000, and two 50 mL aliquots were filtered through a 0.45 µm nitrocellulose filter. The filters were placed on McC agar plates (non-selective McC agar for *Enterobacterales* isolation) and McC + cefotaxime (McC+CTX; 1 mg/L) agar plates (selective agar for the isolation of ESBL or AmpC producing *Enterobacterales*). After 24-hour incubation at 37°C, three typical colonies from both media were subcultured on nutrient agar (NA) and treated as described above. A total of 960 colonies were subjected to species identification. Selection criteria for AST consisted of 1 *K. pneumoniae* isolate per sampling day and per WWTP.

1.3 **Diseased animals**

A total of 242 isolates were obtained through passive surveillance of diseased animals between October 2019 and January 2021 from first line animal health laboratories according to the EARS-Vet scope by Animal Healthcare Flanders, Belgium ^33^. These included pigs (n=137), cattle (n=70), poultry (n=13), and others (n=22). Additionally, 17 *K. pneumoniae* isolates from diseased food-producing animals and a companion animal from January 2020 until September 2021 from Association Régionale de Santé et d’Identification Animales (ARSIA), Belgium, were included in the study. Information on symptoms or antimicrobial treatment was not available. All isolates were screened for presumptive ESBL producers using McC+CTX (1mg/L) plates, and those identified as such were included for AST. Selection for AST of non-ESBL producing isolates was based on animal species (cattle, pig and poultry) and gastrointestinal tract origin, totaling 126 *K. pneumoniae* isolates.

Furthermore, 85 *K. pneumoniae* isolates from individual milk samples from cows with mastitis collected between March and December 2021 were included, comprising clinical (n = 85) and subclinical (n = 3) cases. A total of 58 isolates were retained in the study (9 other species and 18 isolates did not grow after storage). Milk samples (15-25 ml) were collected from one quarter, with 10 µl seeded onto Aesculin Blood Agar and McC agar and incubated at 37°C for 24 hours. Biochemical identification tests were performed, and isolates were confirmed as *K. pneumoniae* by MALDI-TOF MS (Bruker Daltonics).

1.4 **Human isolates**

A total of 100 clinical *K. pneumoniae* isolates resistant to a carbapenem were collected from hospital laboratories and referred to the NRC for voluntary national surveillance. One single isolate per patient corresponding to the timeframe and location of the wastewater sampling campaigns was included.

1.5 **Species identification**

All isolates were identified using MALDI-TOF MS following manufacturer’s instructions (Bruker Daltonics, Bremen, Germany). MBT Compass Library Revision K (April 2019), Revision L (November 2020) and Revision M (July 2021) were used. Isolates identified as *K. pneumoniae* by MALDI-TOF MS will be referred to as presumptive *K. pneumoniae* until species confirmation by WGS.
