# Supplementary material for: One Health genomic surveillance of Klebsiella pneumoniae in Belgium demonstrates large genomic diversity, presence of ESBLs and carbapenemases and circulation of clones across sectors
Source: Front Microbiol. 2026 Oct 7;17:1933141. doi: 10.3389/fmicb.2026.1933141 (PMC13683611; doi:10.3389/fmicb.2026.1933141)
Supplement: Supplementary file 1 [file Data_Sheet_1.zip › Supplementary material/Supplementary material 2_revised.docx]

**Supplementary material 2 : Selection criteria for antimicrobial susceptibility testing**

Isolates identified as *Klebsiella pneumoniae* by MALDI-TOF MS were considered for antimicrobial susceptibility testing (AST). Selection criteria differed according to the sampling sector.

For isolates originating from wastewater treatment plants (WWTPs) and diseased animals, screening for third-generation cephalosporin (3GC) resistance was performed using MacConkey agar supplemented with cefotaxime (McC+CTX). Presumptive ESBL-producing *K. pneumoniae* isolates recovered on McC+CTX were selected for AST. For wastewater, one *K. pneumoniae* isolate per WWTP per sampling day and per isolation medium was selected for AST. For diseased animals, in addition to the isolates selected based on 3GC resistance, 3GC-susceptible isolates were included to obtain a representative selection across the major food-producing animal species (cattle, pigs, and poultry).

For the food-chain sector, isolates were selected to represent major food-producing animal species, including cattle, pigs, and poultry. Selection was further restricted to isolates originating from the intestinal tract.
