# Supplementary material for: One Health genomic surveillance of Klebsiella pneumoniae in Belgium demonstrates large genomic diversity, presence of ESBLs and carbapenemases and circulation of clones across sectors
Source: Front Microbiol. 2026 Oct 7;17:1933141. doi: 10.3389/fmicb.2026.1933141 (PMC13683611; doi:10.3389/fmicb.2026.1933141)
Supplement: Supplementary file 1 [file Data_Sheet_1.zip › Supplementary material/Supplementary material 3REVISED.docx]

Supplementary material 3 - Guidelines used in this study for the interpretation of SNP distances (Luterbach et al., 2023) (Zhang et al., 2022)

| **Threshold** | **Interpretation** |
| --- | --- |
| 0-1 SNPs | Most likely linked |
| 2-5 SNPs | Very likely linked |
| 6-10 SNPs | Likely linked |
| 11-20 SNPs | Possibly linked |
| >20 SNPs | Not linked |

SNP distances should be interpreted in conjunction with epidemiological evidence and should not, on their own, be considered evidence of transmission or epidemiological linkage.

Luterbach, C. L., Chen, L., Komarow, L., Ostrowsky, B., Kaye, K. S., Hanson, B., et al. (2023). Transmission of Carbapenem-Resistant *Klebsiella pneumoniae* in US Hospitals. *Clinical Infectious Diseases* 76, 229–237. doi: 10.1093/cid/ciac791

Zhang, Y., Chen, C., Wu, J., Jin, J., Xu, T., Zhou, Y., et al. (2022). Sequence-Based Genomic Analysis Reveals Transmission of Antibiotic Resistance and Virulence among Carbapenemase-Producing Klebsiella pneumoniae Strains. *mSphere* 7, e00143-22. doi: 10.1128/msphere.00143-22
