# Supplementary material for: One Health genomic surveillance of Klebsiella pneumoniae in Belgium demonstrates large genomic diversity, presence of ESBLs and carbapenemases and circulation of clones across sectors
Source: Front Microbiol. 2026 Oct 7;17:1933141. doi: 10.3389/fmicb.2026.1933141 (PMC13683611; doi:10.3389/fmicb.2026.1933141)
Supplement: Supplementary file 1 [file Data_Sheet_1.zip › Supplementary material/Supplementary material 4.docx]

Supplementary material 4 - Selected reference genomes

| **Sequence type** | **Reference genome RefSeq accession** |
| --- | --- |
| ST11 | GCF_000445405.1 |
| ST13 | GCF_002951635.1 |
| ST147 | GCF_003031345.1 |
| ST15 | GCF_002209405.1 |
| ST152 | GCF_003957455.1 |
| ST307 | GCF_003934185.1 |
| ST39 | n/a* |
| ST392 | GCF_001952875.1 |
| ST42 | GCF_036347675.1 |
| ST4356 | GCF_002257935.4 |
| ST636 | GCF_030845735.1 |
| ST709 | GCF_022353525.1 |
| ST870 | GCF_024918175.1 |
| Novel ST^a^ | GCF_900636455.1 |
| Novel ST^b^ | GCF_002752995.1 |

*(*) The selected reference genome for ST39 was not available on RefSeq, but is available in the on the Intitut Pasteur Paris BIGSdb instance with ID 57203.* This table lists the selected reference genomes for the SNP-based phylogenomic analysis. The first column lists the sequence type. The second column lists the RefSeq accession number for the selected reference genome.
