# Supplementary material for: One Health genomic surveillance of Klebsiella pneumoniae in Belgium demonstrates large genomic diversity, presence of ESBLs and carbapenemases and circulation of clones across sectors
Source: Front Microbiol. 2026 Oct 7;17:1933141. doi: 10.3389/fmicb.2026.1933141 (PMC13683611; doi:10.3389/fmicb.2026.1933141)
Supplement: Supplementary file 1 [file Data_Sheet_1.zip › Supplementary material/Supplementary table 1.docx]

**Supplementary table 1** - EUVSEC 3 sensititre™ plate antibiotics and their epidemiological cut-off values (ECOFF) used for the interpretation of minimal inhibitory concentration values of *Klebsiella pneumoniae*.

| **Antibiotic** | **ECOFF (mg/L)** |  |
| --- | --- | --- |
| Amikacin | 8 |  |
| Ampicillin | 8* |  |
| Azithromycin | 16* |  |
| Cefotaxime | 0.25 |  |
| Ceftazidime | 0.5 |  |
| Chloramphenicol | 16* |  |
| Ciprofloxacin | 0.125 |  |
| Colistin | 2 |  |
| Gentamicin | 2 |  |
| Meropenem | 0.125 |  |
| Nalidixic Acid | 8* |  |
| Sulfamethoxazole | 64* |  |
| Tetracycline | 8 |  |
| Tigecycline | 2 |  |
| Trimethoprim | 2* |  |

*ECOFF of *E. coli* was used since no ECOFF is available for *K. pneumoniae* for this antibiotic.
