# Supplementary material for: One Health genomic surveillance of Klebsiella pneumoniae in Belgium demonstrates large genomic diversity, presence of ESBLs and carbapenemases and circulation of clones across sectors
Source: Front Microbiol. 2026 Oct 7;17:1933141. doi: 10.3389/fmicb.2026.1933141 (PMC13683611; doi:10.3389/fmicb.2026.1933141)
Supplement: Supplementary file 1 [file Data_Sheet_1.zip › Supplementary material/Supplementary table 2.docx]

Supplementary table 2 - EUVSEC 2 sensititre™ plate antibiotics and their epidemiological cut-off values (ECOFF) used for the interpretation of minimal inhibitory concentration values of *Klebsiella pneumoniae*

| **Antibiotic** | **ECOFF (mg/L)** |
| --- | --- |
| Cefepime | 0.125* |
| Cefotaxime | 0.25 |
| Cefotaxime/clavulanic acid | 0.25* |
| Cefoxitin | 8 |
| Ceftazidime | 0.5 |
| Ceftazidime/clavulanic acid | 0.5* |
| Ertapenem | 0.03* |
| Imipenem | 1 |
| Meropenem | 0.125 |
| Temocillin | 8 |

*ECOFF of *E. coli* was used since no ECOFF is available for *K. pneumoniae* for this antibiotic
